# Social Media Authentication and Users’ Assessments of Health Information: Random Assignment Survey Experiment

**DOI:** 10.2196/52503

**Published:** 2024-07-09

**Authors:** Stephen Neely, Kaila Witkowski

**Affiliations:** 1 School of Public Affairs University of South Florida Tampa, FL United States; 2 Department of Public Administration Florida Atlantic University Boca Raton, FL United States

**Keywords:** social media, verification markers, vaccine efficacy, health communication, trust

## Abstract

**Background:**

In an effort to signal the authenticity of user accounts, social networking sites (SNSs) such as Facebook and X, formerly known as Twitter, use visual heuristics (blue checkmarks) to signify whether accounts are *verified*. While these verification badges are generally well recognized (and often coveted) by SNS users, relatively little is known about how they affect users’ perceptions of accuracy or their likelihood of engaging with web-based information. This is particularly true in the case of information posted by medical experts and health care professionals.

**Objective:**

This study aims to use an experimental survey design to assess the effect of these verification badges on SNS users’ assessments of information accuracy as well as their proclivity to recirculate health information or *follow* verified medical experts in their social network.

**Methods:**

A survey experiment using random assignment was conducted on a representative sample of 534 adult SNS users in Florida, United States. A total of 2 separate experimental scenarios exposed users to vaccine-related posts from verified medical experts on X. In each case, the original post contained a platform-issued verification badge (treatment group), which was subsequently edited out of the image as an experimental control. For each scenario, respondents were randomly assigned to either the treatment or control group, and responses to 3 follow-up questions were assessed through a series of chi-square analyses and 2 logit regression models. Responses were fielded using a stratified quota sampling approach to ensure representativeness of the state’s population based on age, sex, race, ethnicity, and political affiliation.

**Results:**

Users’ assessments of information accuracy were not significantly impacted by the presence or absence of verification badges, and users exposed to the experimental treatment (verification badge) were not any more likely to repost the message or *follow* the author. While verification badges did not influence users’ assessments or subsequent behaviors, reliance on social media for health-related information and political affiliation were substantial predictors of accuracy assessments in both experimental scenarios. In scenario 1, which included a post addressing COVID-19 vaccine efficacy, users who relied on social media “a great deal” for health information were 2 times more likely to assess the post as accurate (odds ratio 2.033, 95% CI 1.129-3.661; *P*=.01). In scenario 2, which included a post about measles vaccines, registered Republicans were nearly 6 times *less* likely to assess the post as accurate (odds ratio 0.171, 95% CI 0.097-0.299; *P*<.001).

**Conclusions:**

For health professionals and medical experts wishing to leverage social networks to combat misinformation and spread reliable health-related content, account verification appears to offer little by way of added value. On the basis of prior research, other heuristics and communication strategies are likely to yield better results.

## Introduction

### Overview

Over the past decade, social networking sites (SNSs) such as Facebook and X (formerly known as Twitter) have come to play a central role in the exchange of web-based health information. Evidence collected across a range of geographical contexts showed heavy use of these platforms for information seeking during the COVID-19 pandemic [1-3], and health professionals have increasingly sought to engage with SNS users through these mediums in an attempt to promote timely and reliable health guidance [4,5]. On the one hand, these tools promise greater access and connectivity for patients and consumers, and recent evidence has suggested that individuals infected with COVID-19 were able to gain valuable information and social support through engagement with their SNS communities during the pandemic [6]. On the other hand, social networking platforms such as X have proven highly susceptible to misinformation and politicization, particularly as it relates to the safety and efficacy of vaccines [7,8]. Evidence collected to date suggests that exposure to web-based misinformation has significantly undermined efforts toward vaccine acceptance and had a deleterious impact on public health outcomes [9,10]. Moreover, many SNS users do not make a deliberate effort to integrate reliable, scientific sources into their social networks [2], thereby leaving themselves more vulnerable to unchecked misinformation.

Attempting to balance free-speech protections with a desire to promote accurate and reliable information, SNS platforms have adopted a number of measures to protect and inform users in the face of this ambiguity. In the most drastic cases, these efforts have included third-party or independent fact-checking of posts as well as the deplatforming of users who violate established rules and norms [11,12]. A more subtle and longstanding method has included *authentication* of influential accounts through the use of a visual heuristic (typically a blue checkmark) that accompanies the user’s account profile. While *verification badges* such as the blue checkmark were initially intended to denote the *authenticity* and validity of influential accounts [13], questions have been raised over what, if any, impact these badges have on trust and perceived credibility when it comes to assessments of informational utility [14,15]. While some have argued that the verification of accounts as *authentic* might positively influence assessments of informational credibility, particularly insofar as visual heuristics have been shown to correlate with perceptions of credibility [16], prior studies have failed to find a positive correlation between verification badges and SNS users’ assessments of information credibility. Moreover, the increasing politicization of public health discourse in the United States suggests that, through processes such as hostile media effects and confirmation bias, political identity may be a more influential determinant of perceived credibility than verification badges. The relevance of this question has increased over the past year, as X has made verification contingent upon a monthly subscription fee of US $8 to US $11 [17].

With these considerations in mind, we conducted a survey experiment, using random assignment, to better understand whether verification badges impact users’ perceptions of information quality as well as their likelihood of engaging with health professionals in their social networks. In light of recent events, we designed this survey experiment specifically around posts on vaccine safety and efficacy. While prior research has suggested that verification badges may not improve credibility assessments on the part of information consumers [15], we are unaware of any studies that have examined this relationship specifically in the context of medical experts discussing vaccine efficacy. As health professionals increasingly seek ways to engage with patients and the public in digital spaces, understanding the merits of account verification is an important step. Notably, the verification process is often laborious and bureaucratic in nature, and in the case of X, it now requires a recurring financial commitment. The results of this study are intended to assist health professionals in determining whether account verification is worth the time and expense, while also deepening our understanding of how verification badges affect consumer assessments of information in digital spaces.

### Background Information

While a notable body of literature has considered the factors associated with consumer assessments of information credibility on social media [18-21], relatively little attention has been paid to the impact of account authentication on these assessments. Prior research has indeed found that the metafeatures associated with social media accounts can influence users’ perceptions of their credibility. For instance, the number of followers associated with an account and the frequency with which the account posts content have been found to correlate positively with users’ assessments of the account’s credibility [19]. This is consistent with the finding that cognitive heuristics such as endorsement and persuasive intent are commonly used mechanisms for evaluating information credibility in digital contexts [20].

Moreover, the visual design and presentation of user profiles as well as content attributes have been shown to significantly influence credibility assessments on social media. For example, Morris et al [21] found that perceived credibility was negatively correlated with the use of animated avatars, nonstandard punctuation, and poor grammar. Conversely, the inclusion of multimedia features, such as reliable URLs and infographics, has been found to positively improve credibility assessments and the overall utility of social media posts [22,23]. This latter finding has been particularly salient in the case of web-based health information [22,23]. In addition, Westerman et al [24] found that the “recency” of an account’s posts (ie, the speed with which it provided new content and updates) was positively correlated with perceived source credibility, suggesting that credibility assessments may to some degree be a function of perceived timeliness.

While account authentication, and the associated verification badges such as X*’*s patented blue check, is often coveted by would-be influencers, remarkably little attention has been paid to their role in this complex calculus of credibility assessments. While Morris et al [21] found that SNS users often cite verification as an indicator of credibility when discussing social media in generic terms, experimental studies examining the impact of verification badges have not supported this claim. Among a limited number of studies that have used robust methodologies to directly examine the impact of verification badges, researchers have tended to find that these markers have negligible impacts on credibility assessments. For example, Vaidya et al [13] found that the presence of verification badges did not increase users’ perceptions of credibility when compared with a control group with no indicators of account verification. Moreover, the likelihood that a user would share or recirculate the information contained in a post was not influenced by the presence or absence of a verification badge in their experiment. In a similar study, Edgerly and Vraga [15] found a comparable null effect, noting that “Our results suggest little attention is paid to the verification mark when judging credibility, even when little other information is provided about the account or the content.”

Vaidya et al [13] noted that they were “surprised” that the presence of verification badges did not have an additive effect on users’ perceptions of credibility, and at a glance, their surprise seems warranted. Studies conducted on the uses and gratifications of media have consistently identified learning and utility as primary motivators of media choices and consumption [25,26], and subsequent studies have underscored the relevance of this finding in digital contexts such as social media [27,28]. Viewed through this lens, it is reasonable to assume that the visual authentication of influential accounts might be associated with greater perceived credibility and utility on the part of information consumers. However, there are also reasons to believe that current trends in public health and political discourse might undermine the impact of verification badges on assessments of information credibility.

Chief among these considerations is the potential impact of hostile media effects, whereby individuals are inclined to assess media coverage and information sources as being biased against their predilections, even when the information presented is neutral or scientifically grounded [29,30]. This tendency has been exacerbated by the sharp politicization of public health discourse in the United States over the past decade, which reached a crescendo amid the COVID-19 pandemic [31,32]. For individuals who are inclined to distrust public institutions, such as the media or established government or public health agencies, this could result in a *hostile* response to heuristic cues such as verification badges, which may be interpreted as an endorsement of *establishment norms* on the part of some users rather than as an indicator of authenticity. Over recent years, these tendencies have been further exacerbated by politicized claims that social media companies themselves exercise bias when adjudicating information credibility and promoting subject matter expertise [33,34].

As public health discourse becomes increasingly politicized, the tendency of partisan information consumers toward confirmation bias may also play a significant role in shaping these credibility assessments. Derived from the theory of cognitive dissonance by Festinger [35], research on confirmation bias has underscored the tendency of consumers to selectively avoid information that contradicts their established viewpoints, and to assess such information as biased or unreliable when confronted with counter attitudinal messaging [36,37]. Research conducted during the COVID-19 pandemic has underscored the extent to which these tendencies can lead to dramatically different health behaviors among partisan groups [9,10]. While not explicitly addressed in prior studies, these trends and tendencies may help to explain the results of prior experiments that have found that verification badges do not lead to any improvements in SNS users’ assessments of information credibility.

### Hypotheses

With these considerations in mind, we tested 4 unique hypotheses when conducting this study. On the basis of the findings of prior studies [15,25], we posit a null hypothesis when considering the expected relationship between verification badges and user assessments of information accuracy. As has been found in other contexts, we expect that verification badges will not significantly increase perceptions of information accuracy in the context of vaccine-related posts from verified health experts, which leads to our first hypothesis:

- Hypothesis 1: *Verification badges will not significantly impact SNS users’ assessments of information accuracy.*

In addition, we expect that the presence of a verification badge will not impact the likelihood that SNS users will further engage with social media content, such as reposting it or *following* the authoring account. This too is consistent with the findings of prior research [25], leading to our second hypothesis:

- Hypothesis 2: *Verification badges will not significantly impact the likelihood of SNS users engaging with health-related content on social media.*

Conversely, based on the increasingly politicized nature of public health discourse, particularly as it relates to COVID-19 and other vaccines, we hypothesize that political affiliation will be a significant predictor of SNS users’ assessments when it comes to information accuracy. Throughout the COVID-19 pandemic, in particular, Republican voters have demonstrated significantly higher levels of vaccine hesitancy and less overall trust in public health institutions. Furthermore, recent data have suggested that Republican voters have less confidence in the impartiality and trustworthiness of social media platforms and companies [33,34]. For these reasons, we anticipate that self-identified Republicans will be less inclined to assess provaccine posts from public health experts as accurate than Democratic and independent voters.

- Hypothesis 3: *Political affiliation will be significantly correlated with SNS users’ assessments of information accuracy.*

Finally, we hypothesize a positive correlation between reliance on social media for health information and positive assessments of information accuracy. Recent evidence suggests that SNS users who rely more heavily on social platforms for news and information place a greater premium on utility when managing their information exposure [38-40]. Moreover, to the extent that *reliance* corresponds with greater frequency of *use*, it can be inferred that platform literacy will increase, leading to more accurate assessments of information quality, regardless of visual heuristics.

- Hypothesis 4: *Reliance on social media for health information will be positively correlated with assessments of the accuracy of health-related posts from medical experts.*

## Methods

### Data Collection

Using Prodege MR, an industry-leading market research provider, we conducted a survey of 600 adults in Florida, United States. The survey was sponsored by the Florida Center for Cybersecurity and fielded between August 10, 2023, and August 21, 2023. A stratified quota sampling approach was used to ensure that respondents were representative of the state’s population based on age, sex, race, ethnicity, college education, and political affiliation. For each of these factors, balanced quotas were stratified by region of the state to further ensure representativeness. Sampling quotas were determined based on data from the US Census Bureau and Florida’s Office of Economic and Demographic Research. Among the 600 respondents, 89% (534/600) reported having an active, personal account on social media. Only these respondents are included in this analysis. Table 1 provides a demographic summary of the survey respondents compared with the state’s population parameters.

Participants were required to clear a bot detection test to enter the survey, and a series of quality control questions were dispersed throughout the survey to ensure the highest quality of responses. Respondents who failed to correctly answer quality control questions were removed from the sample; their responses are not included in this analysis and were not counted toward fulfillment of the sampling quotas. On the basis of the sample size and attributes, results were reported with a 95% CI and a margin of error –4 to +4.

**Table 1:** Demographic profile of the survey respondents.

			Active social media users (n=534), n (%)		University of South Florida and Florida Atlantic University survey sample (n=600), n (%)		Florida’s demographics^a^ (%)
**Sex**							
	Female	273 (51.1)		306 (51)		51.10	
	Male	259 (48.5)		292 (48.7)		48.90	
	Intersex	2 (0.3)		2 (0.3)		—^b^	
**Age (years)**							
	18-24	62 (11.6)		64 (10.7)		10.80	
	25-44	186 (34.8)		193 (32.2)		31.20	
	45-64	166 (31.1)		191 (31.8)		32.40	
	≥65	120 (22.5)		152 (25.3)		25.60	
**Race**							
	Black or African American	100 (18.7)		103 (17.2)		16.90	
	White	379 (71)		431 (71.8)		77.30	
	Other	55 (10.3)		66 (11)		5.80	
**Ethnicity**							
	Hispanic	162 (30.3)		174 (29)		26.40	
	Non-Hispanic	372 (69.7)		426 (71)		73.60	
**Education**							
	<4-year degree	360 (67.4)		403 (67.2)		69.50	
	≥4-year degree	174 (32.6)		197 (32.8)		30.50	
**Political affiliation (registered voters only, n=524)**							
	Democrat	190 (35.4)		206 (34.4)		36.20	
	Independent or other	154 (28.9)		174 (29)		28.10	
	Republican	190 (35.6)		220 (36.6)		35.70	
**Region**							
	Panhandle	42 (7.9)		43 (7.2)		7.20	
	Northeast Florida	76 (14.2)		83 (13.8)		12.40	
	Central Florida	130 (24.3)		151 (25.2)		25.50	
	West Coast	119 (22.3)		138 (23)		21.90	
	Southeast Florida	167 (31.3)		185 (30.8)		32.90	

^a^On the basis of the data provided by the US Census Bureau’s 2019 American Community Survey.

^b^Not provided by the US Census Bureau.

### Ethical Considerations

In adherence with ethical standards in research, the survey was approved by the University of South Florida’s institutional review board (#005962). Informed consent was obtained using a standard institutional short form at the outset of the survey. Participants were instructed that by proceeding they were providing their consent to participate and that they could discontinue participation at any point. Discontinuing would mean that their responses were not included in the analysis. A third-party panel vendor was used to obtain access to the survey respondents. No personally identifying information was provided to the researchers by the panel vendor, making the data analyzed for this study deidentified. While participants may have received compensation through the panel vendor, no compensation or benefits for participation were provided by the research team.

### Experimental Design

To examine the impact of verification badges on SNS users’ assessments of informational accuracy as well as their likelihood of engaging with health experts in their social networks, we used 2 separate experimental scenarios. To construct these scenarios, we began by conducting a keyword search of X for posts addressing “vaccine efficacy.” Only posts authored by medical experts with publicly visible and authenticated accounts were selected. From the identified posts, we randomly selected 1 addressing the COVID-19 pandemic for inclusion in the first experimental scenario and a second from non–COVID-19–related posts, which addressed the safety of measles vaccines for the second scenario. The rationale for selecting 1 COVID-19 and 1 non–COVID-19 post was driven by the recent politicization of COVID-19 vaccines [31,32] and a desire to avoid conflating users’ assessments of verification badges with politicized attitudes surrounding the pandemic.

Images of the original posts, containing the blue verification checkmarks, were uploaded to the survey software (Qualtrics XM) as the *treatment* for each scenario. Each image was subsequently edited to remove the blue verification checkmark, and these corresponding images were uploaded as the *control* for each scenario.

For the first experimental scenario, the selected post addressed COVID-19 vaccine effectiveness [41]. The originator of the post is identified in their user profile as a medical doctor with expertise in infectious disease and epidemiology, as well as “an American physician and medical journalist” who was a member of President Joe Biden’s COVID-19 Advisory Board transition team.

Using the randomization feature in Qualtrics, respondents were randomly assigned either the original or edited version of the post and asked to carefully review it before answering subsequent questions. For the purposes of this analysis, respondents randomly assigned to the original image *with* the blue verification checkmark were classified as the *treatment group*, while respondents assigned to the edited image (with the verification badge removed) were classified as the *control group*. While a prior experimental study has suggested that just over half of SNS users notice the verification badge [14], we did not make any additional alterations to the posts or attempt to draw attention to verification badges for the treatment groups. Instead, the badges were presented just as they appear in daily social media use, as the goal of this study is to understand whether these badges have an effect on SNS users’ everyday adjudication of information credibility. Only active social media users were included in the analysis. Thereby, if members of the treatment group failed to notice or recognize the verification badge in responding to the survey, then it stands to reason that they likely fail to do so in their regular SNS use, which suggests that the badges do not routinely impact their assessments of information accuracy.

After being presented with the randomly assigned image, each respondent was asked a series of 3 follow-up questions. The scenarios and questions were designed by the authors uniquely for this study, though they were based on similar experiments used in prior studies [14,15]. The follow-up questions included the following:

- Q1: How confident are you that the information contained in this post is accurate? *(very confident, somewhat confident, not very confident,* and *not at all confident)*
- Q2: If you encountered this post on social media, how likely would you be to repost or “like” it? *(very likely, somewhat likely, not very likely,* and *not at all likely)*
- Q3: How likely would you be to “follow” the person who posted this message? *(very likely, somewhat likely, not very likely,* and *not at all likely)*.

For scenario 2, the selected post spotlighted a recent study that the author said disproved the claim that measles vaccines cause autism [42]. The originator of the post is identified in their user profile as a medical doctor, as well as an “infectious disease physician, coinventor of a rotavirus vaccine, and author” [42]. It should be emphasized that these biographical profiles were not included in the images shown to survey participants, so as not to bias their reactions to the presence or absence of a verification marker. For scenario 2, the same random assignment procedure was followed, and respondents were presented with the same 3 follow-up questions. While each survey participant responded to both of the experimental scenarios, random assignment to the treatment and control groups was made independently for each scenario. It should also be noted that our analysis did not attempt to adjudicate or verify the accuracy of claims made in these posts, as this study is focused solely on SNS users’ reactions to the presence or absence of verification badges.

As outlined in the literature review, several attributes of an account’s user profile and a post’s message content are believed to influence consumer perceptions of information credibility. These can include, but are not limited to, attributes such as username, profile image, grammar, punctuation, and the inclusion of multimedia content such as infographics and URLs. It is worth pointing out that several of these attributes may be factors in overall assessments of the posts included in this analysis. However, the goal of this study is not to measure or explain aggregate assessments of information accuracy. Instead, we are narrowly focused on whether the presence of verification badges for authenticated accounts influences these assessments. In other words, our only concern in this instance is the observed variation between the treatment and control groups, and because these attributes do not vary across the treatment and control groups, they cannot explain any such variation. Therefore, we do not attempt to assess the impact of these attributes on participants’ assessment of, or willingness to engage with, the posts included in either experimental scenario. In addition, factors such as individual health literacy and familiarity with the included accounts are not considered in this analysis, though the experimental design used by this study ensures that variations in such factors will be randomly distributed across the treatment and control groups.

### Statistical Analysis

Because this study uses random assignment, which distributes demographic characteristics and innate behavioral tendences randomly across groups, we began our analysis with a series of simple chi-square tests to examine differences in response tendencies across the treatment and control groups (Stata 17; StataCorp LLC). After this, we estimated 2 logistic regression models to test hypotheses 3 and 4 (Stata 17). In these models, we focus specifically on the first follow-up question for each scenario: *How confident are you that the information contained in this post is accurate?* For the purposes of these models, the “very confident” and “somewhat confident” responses were recoded as “confident” (confident=1), while the “not very confident” and “not at all confident” responses were recoded as “not confident” (not confident=0). While an ordered logit model would be ideal for this analysis given the ordinal nature of the outcome variable, our data would not support the cell-size assumptions associated with this technique due to the large number of categorical predictor variables. The 2 logit models were estimated as shown in equations 1 and 2:



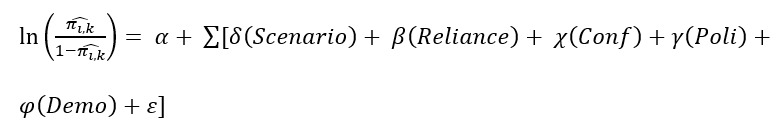

**(1)**




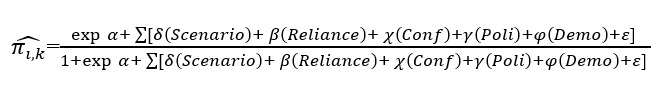

**(2)**


Where 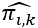 is the estimated probability that the i^th^ case indicated *confidence* in informational accuracy for scenario k; *Scenario* indicates whether the respondent was randomly assigned to the treatment or control group; *Reliance* is a measure of how heavily the respondent relies on social media for health-related information; *Confidence* is a measure of the respondent’s self-reported confidence in public health officials; *Poli* is a measure of the respondent’s political affiliation, and *Demo* is a vector of demographic control variables, including age, sex, race, ethnicity, and education.

Respondents assigned images with the blue verification checkmark were coded as the *treatment* group (treatment=1), while those assigned images without the verification markers were coded as the *control* group (control=0). The *Reliance* variable was measured ordinally based on responses to the following prompt: *Over the past 3 years, how heavily have you relied on social media to stay up-to-date and informed about the COVID-19 pandemic?* Response options included: “a great deal” (108/534, 20.2%), “a little” (195/534, 36.5%), “not very much” (157/534, 29.4%), and “not at all” (74/534, 13.9%). The *Confidence in Public Health Officials* variable was measured ordinally as well, based on the following prompt: *Please indicate to what extent you trust the following institutions to operate in the best interests of society...Public Health Officials*. Response options included: “trust a lot” (86/434, 16.1%), “trust to a degree” (261/534, 48.9%), “don’t really trust” (104/534, 19.5%), “don’t trust at all” (63/534, 11.8%), and “unsure” (20/534, 3.7%).

For the *Poli* variable, which measured respondents’ political affiliation, independents and those who selected “third-party or other” were combined due to the small number of respondents who selected “other” (19/534, 3.6%). Democrats were excluded as the reference category in the logit models. While a number of possible variables could be used to measure political identity, we chose to focus on partisan affiliation based on its recognized importance as a predictor of political behavior [43,44] as well as its observed importance in measures of public health behavior, particularly during the COVID-19 pandemic [9,32]. *Age* was log-transformed for the purposes of this analysis, and *Race* was recoded as “White,” “African American,” and “Other” due to sample size constraints. White participants were excluded as a reference category. Non-Hispanic participants, male participants, and those with a <4-year degree were excluded as reference categories, for their respective variables, and those who selected “non-binary” for sex were excluded from the regression model due to sample size (n=2).

## Results

### Scenario 1

Table 2 shows a comparison of response frequencies for each of the 3 follow-up questions in scenario 1. No significant differences were observed across the treatment and control groups for assessments of information accuracy. In addition, no significant differences were observed for either of the engagement questions, suggesting that individuals exposed to the image containing a verification badge were no more likely than the control group to repost or *like* the post or to *follow* the health expert who posted it. In each case, only a small proportion of respondents in each group indicated that they would be “very” or “somewhat likely” to follow the health expert sharing the information (47/268, 17.6% in the control group and 54/266, 20.3% in the treatment group).

Table 3 summarizes results of the logit regression model for scenario 1, examining factors associated with positive assessments of the accuracy of the post. After accounting for reliance on social media, trust in public health experts, political affiliation, and demographic differences, exposure to the verification badge did not have a statistically significant impact on assessments of information quality, ceteris paribus. While those in the treatment group were slightly more likely to assess the information as accurate, the effect size was negligible (odds ratio 1.089, 95% CI 0.747-1.588), and the observed relationship was not statistically significant (*P*=.65).

Reliance on social media for health information, trust in public health officials, and political affiliation were all significantly related to positive assessments of the post’s accuracy. Republicans were nearly 2 times *less* likely than Democrats to assess the information contained in the post as accurate (1/0.570=1.75), ceteris paribus.

Unsurprisingly, trust in public health officials was the most substantial predictor of a positive information accuracy assessment, with those who indicated that they trust public health officials “a lot” being 14 times *more* likely to assess the post as accurate than those who said they did not trust public health officials at all. In addition, high levels of reliance on social media for health information were positively related to assessments of information accuracy, with those who relied on social media “a great deal” being nearly 2 times *more* likely to assess the post as accurate than those who did not rely on social media for health information at all (e^b^=1.873). Notably, there was a greater likelihood to assess the post as accurate among those who relied on social media even “a little” for health information, but this difference was only statistically significantly at the .05 level for those who reported relying on social media “a great deal.”

**Table 2:** Scenario 1: effects of verification badges on the perceptions of information accuracy and subsequent engagement (n=534).

		Control (no verification marker; n=268), n (%)	Treatment (verification marker; n=266), n (%)
**How confident are you that the information contained in the post is accurate?^a^**			
	Very confident	53 (19.8)	55 (20.7)
	Somewhat confident	95 (35.5)	100 (37.6)
	Not very confident	81 (30.2)	76 (28.6)
	Not at all confident	39 (14.6)	35 (13.2)
**If you encountered this post on social media, how likely would you be to repost or “like” it?^b^**			
	Very likely	34 (12.7)	29 (10.9)
	Somewhat likely	38 (14.2)	51 (19.2)
	Not very likely	80 (29.9)	73 (27.4)
	Not at all likely	116 (43.3)	113 (42.5)
**How likely would you be to “follow” the person who posted this message?^c^**			
	Very likely	12 (4.5)	15 (5.6)
	Somewhat likely	35 (13.1)	39 (14.7)
	Not very likely	89 (33.2)	90 (33.8)
	Not at all likely	132 (49.3)	122 (45.9)

^a^*χ*^2^_3_=0.5; *P*=.91.

^b^*χ*^2^_3_=2.6; *P*=.44.

^c^*χ*^2^_3_=0.9; *P*=.81.

**Table 3:** Logistic regression: scenario 1 (social networking site users’ assessments of accuracy).

		Odds ratio (95% CI)	SE (robust)	*P* value
Experimental group (1=treatment)		1.089 (0.747-1.588)	0.209	.65
**Reliance on social media for health information (reference: not at all)**				
	Not very much	0.964 (0.578-1.607)	0.252	.88
	A little	1.627 (0.969-2.733)	0.431	.06
	A great deal	1.873 (1.016-3.453)	0.125	.04
**Trust in public health officials** **(reference:** **don’t trust at all)**				
	Don’t really trust	2.794 (1.377-5.669)	1.009	.004
	Trust to a degree	5.496 (2.883-10.478)	1.809	<.001
	Trust a lot	14.222 (6.291-32.151)	5.919	<.001
	Unsure	2.364 (0.777-7.195)	1.342	.13
**Political affiliation (reference:** **Democrat)**				
	Independent or Other	0.808 (0.475-1.373)	0.219	.43
	Republican	0.570 (0.333-0.978)	0.157	.04
	Nonvoter	0.566 (0.296-1.081)	0.187	.08
Age (log-transformed)		0.899 (0.701-1.154)	0.114	.40
**Sex (reference: male)**				
	Female	0.952 (0.640-1.416)	0.193	.80
**Race (reference:** **White)**				
	African American or Black	0.605 (0.352-1.042)	0.168	.07
	Other	0.409 (0.210-0.796)	0.139	.008
Hispanic (1=yes)		1.176 (0.742-1.863)	0.276	.49
4-year degree (1=yes)		1.262 (0.834-1.908)	0.266	.27
Constant		0.442 (0.131-1.494)	0.266	.18
−2 log likelihood		–318.920 (—^a^)	—	—
Pseudo *R*^2^		0.124 (—)	—	—

^a^Not applicable.

### Scenario 2

Table 4 shows a comparison of response frequencies for each of the 3 follow-up questions in scenario 2. No statistically significant differences were observed across the control and treatment groups’ assessment of the post’s accuracy. In addition, no significant differences were observed in the likelihood of following the medical expert who authored the post. Once again, less than a quarter of respondents in either group indicated that they would be likely to follow the author of the post (54/267, 20.1% in the control group and 63/267, 23.6% in the treatment group).

In the case of reposting or *liking* the post, a statistically significant difference was observed between the treatment and control groups (*P*=.04), though the difference was relatively nuanced. Respondents in the treatment group were *more* likely to say that they would be “very likely” to repost the post (32/267, 11.9% compared to 18/267, 6.7% in the control group). Comparatively, respondents in the control group were *more* likely to say they would be “somewhat likely” to do the same (63/267, 23.6% compared to 43/267, 16.1% in the treatment group). However, when the “very likely” and “somewhat likely” responses were combined, respondents in the control group were *more* likely overall to indicate that they would repost the post (81/267, 30.3% in the control group compared to 75/267, 28.1% in the treatment group). While the observed differences in magnitude are statistically significant, they do not appear to represent a practically meaningful difference in responses.

Table 5 summarizes the results of the logit regression model for scenario 2. Once again, there was no significant difference associated with the verification badge, ceteris paribus. Respondents in the treatment group were slightly *more* likely to assess the post as accurate after controlling for other predictors (odds ratio 1.319, 95% CI 0.877-1.986), but the difference was not statistically significant.

**Figure 1:**
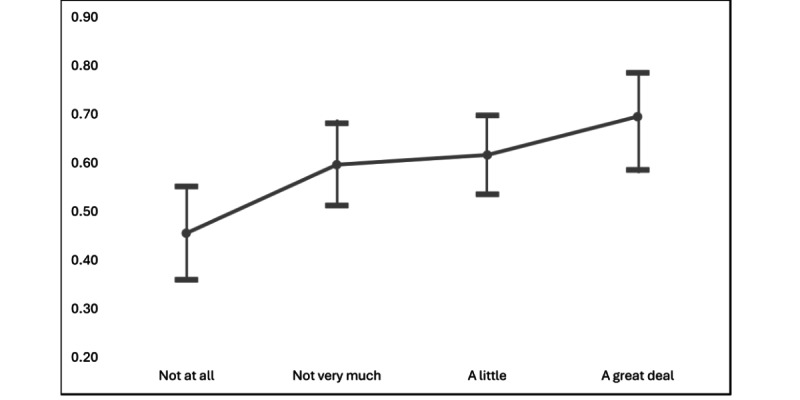
Scenario 1: marginal effects of reliance on social media on the probability of assessing post as accurate.

As in scenario 1, reliance on social media for health information was significantly related to assessments of accuracy, as was trust in public health officials and political affiliation. In the case of political affiliation, a similar pattern to that observed in scenario 1 was seen in scenario 2, though the magnitude was more notable. Specifically, registered Republicans were nearly 4 times *less* likely than registered Democrats to assess the post as accurate (1/0.253=3.95). Those who relied on social media “a great deal” for health information were 2.66 times *more* likely to assess the post as accurate than those who did not rely on social media at all. Figure 1 shows that the marginal probability of assessing the post as accurate increased consistently and significantly as reliance on social media for health information increased.

**Table 4:** Scenario 2: effects of verification badges on the perceptions of information accuracy and subsequent engagement (n=534).

		Control (no verification marker; n=267), n (%)	Treatment (verification marker; n=267), n (%)
**How confident are you that the information contained in the post is accurate?^a^**			
	Very confident	39 (14.6)	41 (15.4)
	Somewhat confident	107 (40.1)	119 (44.6)
	Not very confident	84 (31.5)	71 (26.6)
	Not at all confident	37 (13.9)	36 (13.5)
**If you encountered this post on social media, how likely would you be to repost or “like” it?^b^**			
	Very likely	18 (6.7)	32 (11.9)
	Somewhat likely	63 (23.6)	43 (16.1)
	Not very likely	77 (28.8)	73 (27.3)
	Not at all likely	109 (40.8)	119 (44.6)
**How likely would you be to “follow” the person who posted this message?^c^**			
	Very likely	16 (5.9)	17 (6.4)
	Somewhat likely	38 (14.2)	46 (17.2)
	Not very likely	83 (31.1)	78 (29.2)
	Not at all likely	130 (48.7)	126 (47.2)

^a^*χ*^2^_3_=1.7; *P*=.61.

^b^*χ*^2^_3_=8.2; *P*=.04.

^c^*χ*^2^_3_=1.0; *P*=.80.

**Table 5:** Logistic regression: scenario 2 (social networking site users’ assessments of accuracy).

		Odds ratio (95% CI)	SE (robust)	*P* value
Experimental group (1=treatment)		1.319 (0.877-1.986)	0.275	.18
**Reliance on social media for health information** **(reference: not at all)**				
	Not very much	1.969 (1.105-3.508)	0.580	.02
	A little	2.049 (1.178-3565)	0.579	.01
	A great deal	2.659 (1.394-5074)	0.876	.003
**Trust in public health officials** **(reference:** **don’t trust at all)**				
	Don’t really trust	3.098 (1.389-6.913)	1.269	<.001
	Trust to a degree	9.556 (4.458-20.484)	3.718	<.001
	Trust a lot	20.850 (8.211-52.947)	9.914	.006
	Unsure	3.190 (0.873-11.652)	2.108	.08
**Political affiliation** **(reference: Democrat)**				
	Independent or Other	0.608 (0.335-1.105)	0.185	.10
	Republican	0.253 (0.137-0.468)	0.079	<.001
	Nonvoter	0.242 (0.123-0.476)	0.084	<.001
Age (log-transformed)		0.683 (0.527-0.885)	0.090	.004
**Sex** **(reference: male)**				
	Female	0.857 (0.558-1.316)	0.188	.48
**Race** **(reference: White)**				
	African American or Black	0.736 (0.400-1.355)	0.229	.32
	Other	0.869 (0.464-1.626)	0.278	.66
Hispanic (1=yes)		1.065 (0.657-1.725)	0.262	.79
4-year degree (1=yes)		1.504 (0.962-2.349)	0.342	.07
Constant		0.748 (0.207-2.698)	0.489	.65
−2 log likelihood		–284.935 (—^a^)	—	—
Pseudo *R*^2^		0.216 (—)	—	—

^a^Not applicable

## Discussion

### Overview

Prior research has suggested that SNS verification badges, such as X’s patented “blue checkmark,” may have little to no effect on users’ assessments of informational value [14,15]. We conducted a survey experiment analysis using random assignment of survey respondents to test this hypothesis specifically in the context of posts from verified medical experts addressing vaccine efficacy and safety. In addition, we examined whether the presence or absence of verification badges (in otherwise identical posts) was correlated with differences in the likelihood that SNS users would engage with the posts or add the authors to their social network. Finally, we compared the effects of verification makers with factors such as political affiliation, trust in public health guidance, and reliance on social media for health information. Collectively, the results suggest that verification badges do not have a significant impact on users’ assessments of information accuracy, their likelihood of reposting content, or their likelihood of *following* medical experts.

Reiterating the research design outlined in the *Methods* section, our analysis tested the following 4 hypotheses:

- Hypothesis 1:*Verification badges will not significantly impact SNS users’ assessments of information accuracy.*
- Hypothesis 2: *Verification badges will not significantly impact the likelihood of SNS users engaging with health-related content on social media.*
- Hypothesis 3: *Political affiliation will be significantly corelated with SNS users’ assessments of information accuracy.*
- Hypothesis 4: *Reliance on social media for health information will be positively correlated with assessments of the accuracy of health-related posts from medical experts.*

Each of these hypotheses was confirmed by the analysis. Both a chi-square analysis of differences across the treatment and control groups as well as subsequent logit regression models showed that verification badges did not have a statistically or practically significant impact on SNS users’ assessments of information accuracy. In both scenarios, a similar number of respondents in both the treatment and control groups indicated that they were either “very” or “somewhat confident” that the information contained in the post was accurate. For the first scenario, 55.2% (148/268) in the control group and 58.3% (155/266) in the treatment group adjudicated the post as accurate. In the second scenario, 54.7% (146/267) and 59.9% (160/267) said the same, respectively. In both cases, the observed differences were not statistically significant. Moreover, verification badges did not significantly alter the likelihood that respondents would *follow* the author of the post. While there was a statistically significant difference associated with verification badges and the likelihood of reposting or *liking* the post in 1 of the 2 experimental scenarios, the magnitude of the observed difference was negligible in practical terms.

Notably, respondents in both the treatment and control groups reported a very low propensity to follow the original author of the post in either scenario. For scenario 1, only 17.5% (47/268) of respondents in the control group and 20.3% (54/266) of respondents in the treatment group indicated that they would be at least “somewhat likely” to follow the author of the post. Similarly, in scenario 2, 20.2% (54/267) of respondents in the control group and 23.6% (63/267) of respondents in the treatment groups said the same. Even at the peak of the COVID-19 pandemic, many SNS users had opted not to incorporate expert medical and scientific sources into their social networks, despite relatively high levels of reliance on SNS platforms for pandemic-related information [2]. While many users may view social networks primarily as a tool for connecting with family and social acquaintances, as reliance on these platforms for health information increases [1-3], integrating reliable sources of medical and scientific information may be a necessary adjustment to offset the effects of ambiguity and misinformation.

Consistent with the *hostile media effects* and *confirmation bias* literature, hypothesis 3 posited that political affiliation would be significantly correlated with users’ assessments of informational accuracy. This was supported by the logit regression models, where we found that when compared to registered Democrats, Republicans were between 1.8 and 3.9 times *less* likely to express confidence in the accuracy of the medical posts across the 2 scenarios, respectively. Democrats were also significantly *more* likely than registered independents and nonvoters to perceive the posts as accurate in both experimental scenarios. These findings are consistent with patterns of ideological polarization observed throughout the COVID-19 pandemic, particularly a greater tendency on the part of Democrats to support vaccination efforts and greater belief in vaccine-related misinformation among Republican voters [32,45]. A recent study examining excess mortality rates among registered Democrats and Republicans found that this partisan confirmation bias has had significant real-world implications, most notably that “the excess death rate among Republican voters was 43% higher than the excess death rate among Democratic voters” *after* the introduction of COVID-19 vaccines [46].

Finally, we also found support for hypothesis 4, which posited that greater reliance on social media for health information would be positively associated with assessments of information accuracy. This hypothesis was supported by the findings, and for both scenarios, the marginal probability that respondents interpreted the post as accurate went up notably as their reliance on social media for health information increased (Figure 1). Several recent studies have shown that SNS users who view their social networks as a source of news and information place a greater premium on information utility than others [38-40]. Platform literacy may also be a factor in this relationship, as those who rely on and use SNS sites more heavily are likely to become better adept at adjudicating the quality of information on those platforms.

As health professionals seek ways to effectively leverage emerging technologies in an effort to reach patients and address viral misinformation, the evidence presented here indicates that verification badges offer little added value in terms of perceived expertise and network expansion. While our study does not speak to what, if any, value SNS users might place on *authentication* apart from assessments of accuracy [13], our findings suggest that the time and financial investments associated with obtaining and maintaining account verification will likely yield little benefit in terms of web-based reach and influence. Comparatively, cultivating proven social media strategies and literacy may offer a more impactful approach for health professionals (at both the individual and organizational levels) who wish to expand their influence in these informational spaces.

For example, prior research has indicated that the use of supplemental graphics in posts containing medical information and public health guidance can be an effective avenue for combating misinformation and increasing user perceptions of credibility [16,23,47]. The evolving functionalities of platforms such as Facebook and X now also allow health professionals to engage in proactive outreach efforts, such as live, hosted question and answer sessions to address misinformation and provide real-time fact-checking. As an example, during the Zika virus outbreak, the US Department of Health and Human Services hosted digital town halls on SNS platforms to address questions submitted by SNS users [48]. These efforts may do more to increase reach and influence than verification badges in light of the findings outlined in the *Results* section above. Finally, developing institutional policies [49] and educational programs addressing [50] social media use by health professionals can help to optimize the use of these platforms for outreach, while also helping health professionals to overcome some common barriers to adoption of these increasingly critical technologies [49,51].

### Limitations and Future Research

There are some natural limitations associated with web-based survey experiments, such as the one conducted in this study. While the sample of respondents is highly representative of the target population parameters based on standard demographic factors (such as age, sex, race, ethnicity, and political affiliation), some important population groups are likely to be underrepresented by this methodology. These include, but are not necessarily limited to, those without a high school diploma, members of rural communities that lack reliable internet access, non-English speakers, residents of nursing homes, and individuals who were incarcerated or institutionalized. These factors should be kept in mind when interpreting the findings.

Moreover, our study focuses specifically on the effect of verification badges on a single platform (X). Differences in user populations, social norms, political tendencies, and functionality across platforms may impact results, including how users interpret the value and meaning of verification badges. Future studies should consider these relationships across platforms that use the same format for verification badges, such as Facebook and Instagram. In addition, the focus on vaccine-related content in these experimental scenarios, while germane to current public health discourse, may introduce a level of controversy and politicization that could influence user’ responses. We recommend that additional studies consider a more diverse range of medical topics when adjudicating the value of verification badges for medical posts and authors.

Finally, as part of a larger survey on health policy and administration in Florida, our analysis was somewhat limited by constraints on questionnaire length. However, some additional measures might help to deepen our understanding of how users interpret verification badges. These include individual health literacy, experience, and anxiety, as well as the respondent’s self-reported level of trust in social media platforms as a source of information (distinct from their reliance on these platforms for information) and their perceptions of SNS platforms as a vehicle for *context collapse* [52] as opposed to a tool for enhancing existing interpersonal relationships. We recommend that future studies consider these factors to deepen our understanding of this important phenomena.

## Abbreviations

SNS: social networking site
